# L-Theanine Mitigates Chronic Alcoholic Intestinal Injury by Regulating Intestinal Alcohol and Linoleic-Arachidonic Acid Metabolism in Rats

**DOI:** 10.3390/nu17111943

**Published:** 2025-06-05

**Authors:** Jiayou Gu, Simin Tan, Jiahao Yang, Xuhui Dang, Kehong Liu, Zhihua Gong, Wenjun Xiao

**Affiliations:** 1 National Research Center of Engineering and Technology for Utilization of Botanical Functional Ingredients, Hunan Agricultural University, Changsha 410128, China; 18175550605@163.com (J.G.); 16673431436@163.com (S.T.); 15616941136@163.com (J.Y.); 18991932097@163.com (X.D.); sumzzzz@163.com (K.L.); gzh041211@163.com (Z.G.); 2 Key Laboratory of Tea Science of Ministry of Education, Hunan Agricultural University, Changsha 410128, China

**Keywords:** alcohol consumption, chronic alcoholic intestinal injury, L-theanine, alcohol metabolism, linoleic-arachidonic acid metabolism

## Abstract

**Background**: Chronic alcohol intake impairs intestinal function, while L-theanine (LTA) may support intestinal health. However, the protective effects of LTA to chronic alcoholic intestinal injuries remain unclear. **Methods**: SD rats were administered LTA for 8 weeks and then co-administered Lieber–DeCarli liquid alcohol feed and LTA for 4 weeks to establish a chronic alcoholic intestinal injury model and investigate the mitigating influence of LTA on chronic alcoholic intestinal injury. **Results**: LTA alleviated duodenal pathology and intestinal permeability injury and reduced intestinal oxidative stress and inflammatory response, thereby mitigating chronic alcoholic intestinal injury. Additionally, LTA ameliorated disturbances in the gut microbiota induced by chronic alcohol intake by increasing the beneficial bacteria abundance (*Ruminococcus* and *Odoribacter*) and decreasing the harmful bacteria abundance (*Enterococcus*). Moreover, LTA altered the metabolic profiles associated with ethanol and linoleic (LA) and arachidonic acid (AA) metabolism. ADH6, ALDH2, and ACSS1 mRNA and protein levels were upregulated by LTA, whereas those for CYP2E1, FADS2, ALOX-5, and COX-1 were downregulated. Concurrently, LTA increased the levels of metabolites, such as acetyl-CoA, and decreased the levels of ethanol, acetaldehyde, acetic acid, LA, AA, PGE2, 13-HPODE, and LTB4. **Conclusions**: L-theanine mitigates chronic alcoholic intestinal injury by regulating intestinal alcohol and LA-AA metabolism. Our findings support the functional potential of the dietary supplement LTA and highlight its potential for addressing chronic intestinal injury caused by chronic alcohol intake.

## 1. Introduction

Alcohol stands as a widely favored drink, but its excessive consumption can lead to various health issues. Chronic alcohol abuse poses a significant global public health challenge [1,2]. The intestine is the main organ of alcohol absorption. Most studies on the effects of alcohol consumption have focused on alcohol-related liver conditions. However, the adverse effects of alcohol on the intestine, the main site of absorption, should also be considered. A recent comprehensive study showed that the intestinal tract accelerates the clearance of acetaldehyde and makes a non-negligible contribution to alcohol metabolism [3]. Alcohol-induced intestinal injury is partly caused by the toxic effects of acetaldehyde, which is primarily produced by the alcohol dehydrogenase family (ADHs). Acetaldehyde is metabolized into acetic acid by acetaldehyde dehydrogenases (ALDHs), with ALDH2 being identified as the principal mitochondrial enzyme responsible for eliminating acetaldehyde [4]. Subsequently, acetic acid is converted into acetyl-CoA by acetyl-CoA synthetase, allowing it to participate in the tricarboxylic acid cycle [5]. In instances of excessive alcohol consumption, cytochrome P450 2E1 (CYP2E1) also participates in metabolizing a portion of ethanol into acetaldehyde [6]. However, this process often results in the overactivation of CYP2E1 and leads to increased reactive oxygen species (ROS) production, thereby inducing oxidative stress in the intestine [7].

Alcohol-induced intestinal damage manifests not only as oxidative stress but also as severe immune dysregulation. Chronic alcohol intake leads to the production of pro-inflammatory cytokines, such as tumor necrosis factor-α (TNF-α), interleukin-1β (IL-1β), and interleukin-6 (IL-6), through Toll-like receptor 4 (TLR4)-dependent recognition of intestinal endogenous endotoxin (LPS) [8,9]. At the same time, alcohol inhibits anti-inflammatory pathways, including regulation of IL-10 signaling, and disrupts mucosal immune homeostasis [10]. Approximately 10^14^ bacteria inhabit the intestine, playing a prominent role in regulating the intestinal inflammatory response and maintaining the intestinal barrier [11]. Notably, regulating intestinal metabolism disruption can protect the intestine. Research indicates that unsaturated fatty acids, such as linoleic (LA) and arachidonic (AA) acids, play an important role in this process. Intestinal microbes can convert LA into conjugated AA, thereby bolstering intestinal immunity [12]. Immune dysregulation is exacerbated by metabolic disturbances in the linoleic acid (LA)–arachidonic acid (AA) pathway. AA-derived eicosanoids (PGE_2_, LTB_4_) are potent proinflammatory mediators [13]. In addition, LA is converted into AA by fatty acid desaturase 2 (FADS2), and metabolic AA derivatives, such as prostaglandins (PGs) and leukotrienes (LTs), often exhibit pro-inflammatory properties in the intestine [14,15].

Numerous naturally active ingredients derived from plants, including L-theanine (LTA) [16], garlic fructans [17], and baicalin [18], have been extensively investigated for their potential to alleviate intestinal injury owing to their low toxicity. LTA is a unique amino acid found in tea that is easily absorbed by the intestine and considered safe [19,20]. LTA has a similar structure to glutamine and glutamate, and its biosynthesis is derived from glutamate. It has multiple biological functions, including antioxidant and anti-inflammatory functions [21,22]. In previous studies, our research team has demonstrated that LTA maintains homeostasis within the intestinal microbiome [16,23]. Additionally, our findings indicated that LTA plays a significant role in regulating alcohol metabolism [5]. However, whether LTA protects against chronic alcoholic intestinal injury and its underlying mechanisms remains unknown. Consequently, we established a model of chronic alcoholic intestinal injury in rats. Inflammatory factors, antioxidant enzyme activity, intestinal permeability, and histopathology were used to evaluate the effects of LTA on chronic alcoholic intestinal injury. 16S ribosomal RNA (16S rRNA) gene sequencing and non-targeted metabolomics were used to reveal potential mechanisms of action. These findings were further verified using quantitative real-time polymerase chain reaction (qPCR), Western blotting, and targeted metabolite analyses. This study aims to provide a new strategy for the protection and mitigation of chronic alcohol-induced intestinal injury and provide scientific support for developing LTA as a nutritional supplement.

## 2. Materials and Methods

### 2.1. Reagents

The main reagents in this study are presented in Table S1.

### 2.2. Animals

Fifty male SPF SD rats (5 weeks old, 200–220 g weight) were obtained from Hunan Slack Jingda Laboratory Animal Co., Ltd. (Changsha, China). The study was conducted following NIH guidelines (No. 85-23 Rev. 1985) and the Animal Health and Ethics Committee of Hunan Agricultural University (Approval No. 2022 No. 11). Rats were fed on a 12 h light/dark cycle, with 55% plus or minus 5% humidity and a temperature of 25 °C plus or minus 2 °C in a controlled environment.

### 2.3. Establishment of Chronic Alcoholic Intestinal Injury Model in Rats

Following a one week adaptation period to the liquid diet, chronic alcoholic intestinal injury was induced using a modified version of the Lieber–DeCarli models [24,25]. Specifically, rats were maintained on a Lieber–DeCarli diet supplemented daily with ethanol (12 g/kg body weight) for 4 weeks.

### 2.4. Treatments

After a one week acclimatization period on the liquid diet, the rats were randomly divided into five groups (n = 10): control (CK), model control (MOD), LTA100, LTA200, and LTA400 groups. Rats in the LTA100 group were gavaged LTA at doses of 100 mg·kg^−1^·d^−1^, and rats in the LTA200 and LTA400 groups were gavaged 200 and 400 mg·kg^−1^·d^−1^ LTA, respectively, from 9:00 a.m. to 10:00 a.m. daily for 8 weeks. The dosage of LTA was determined based on previous studies [21].

**Figure 1:**
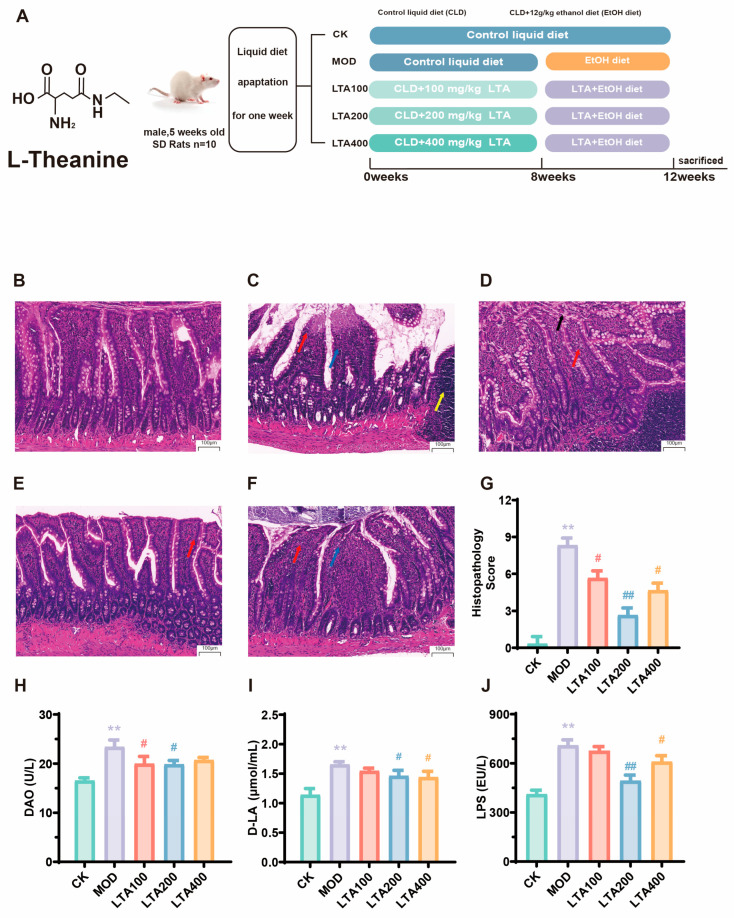
LTA affects the histopathological damage and intestinal permeability of the duodenum in rats. (**A**) Animal grouping and experimental treatment. H&E tissue section of (**B**) CK, (**C**) MOD, (**D**) LTA100, (**E**) LTA200, and (**F**) LTA400 group (H&E, ×20, n = 3). The tips of the villi autolysis (blue arrow), epithelial cells of intestinal villi shed (black arrow), inflammatory infiltration (red arrow), and hyperplasia of lymphoid tissue (yellow arrow). (**G**) The intestinal histopathological injury score (n = 3). (**H**–**J**) Serum diamine oxidase (DAO), D-lactic acid (D-LA), and lipopolysaccharides (LPS) content in rats (n = 6). Compared with CK, **: *p* < 0.01; compared with MOD, #: *p* < 0.05, ##: *p* < 0.01. Data are presented as the mean ± SD.

The BSA conversion factor for SD rats was 0.16 (using a 60 kg human as a reference). The doses of 100, 200, and 400 mg·kg^−1^·d^−1^ in rats matched human equivalent doses of 16, 32, and 66 mg·kg^−1^·d^−1^ [26]. It has been shown that supplementation with 800 mg·kg^−1^·d^−1^ LTA is safe in SD rats, and therefore, we believe that the dose of LTA used in this study is reasonable and safe. The feeding amount of LTA was calculated based on the rats’ body weight and feed intake within each group (Figure S1). Subsequently, a model of chronic alcoholic intestinal injury was constructed at 8–12 weeks, and the rats in the LTA group received both alcohol and LTA. The grouping and treatment of the animals are shown in Figure 1A. On the 84th day, the rats were fasted for 12 h after intervention, and fresh dejection was collected. No adverse occurrences were observed throughout the trial phase. Then, with 2% pentobarbital sodium anesthesia in rats, abdominal aortic blood was collected. We collected the intestinal contents and different intestinal tissues, and the serum was promptly extracted by centrifuging blood samples at 3000 rpm for 10 min at 4 °C.

### 2.5. Histopathological Assessment

Duodenal samples of rats were fixed in 10% formaldehyde solution. Following standard processing, hematoxylin and eosin (H&E) staining was conducted by Pinofey Biotechnology Co., Ltd. (Wuhan, China). Histological evaluation was conducted using modified scoring criteria adapted from previous studies [27], as detailed in Supplementary Table S2.

### 2.6. Biochemical Assessment of Serum and Intestinal Tissues

The levels of diamine oxidase (DAO, AJ-2964A), lipopolysaccharides (LPS, AJ-3418), and D-lactic acid (D-LA, AJ-6386A) in the serum, and the levels of MDA (A003-1-2), superoxide dismutase (SOD, A001-3-2), glutathione peroxidase-px (GSH-Px, A005-1-2), catalase (CAT, A007-1-1), and reactive oxygen species (ROS, E004-1-1) in the small intestinal were determined by ELISA kits. The level of TNF-α (P6335), IL-1β (P6245), IL-6 (P6276), and interleukin-10 (IL-10, P6290) in the small intestine were determined by ELISA kits. The ELISA kits were all obtained from Shanghai Biyontime Biotechnology Co., Ltd., (Shanghai, China). All of the above indicators were detected by an EL×800 enzyme labeling instrument (BioTek Instruments, Inc., Winooski, VT, USA), and all indicators were strictly early in accordance with the kit instructions, and the batch-to-batch CV of all indicators was ≤6%.

### 2.7. 16S rRNA-Based Intestine Microbiota Determination

Fecal samples from rats were collected, and genomic DNA was extracted using the E.Z.N.A. genomic DNA extraction kit (Omega Bio-Tek, Norcross, GA, USA). The detection and analysis methods followed previously described protocols [16].

### 2.8. LC/MS-Based Intestine Metabolomics

The intestinal contents of rats were collected, and the samples were processed and detected according to established protocols [23]. The screening criteria were VIP > 1 and *p*-value < 0.05. Metabolic pathway enrichment analysis was performed using MetaboAnalyst 5.0 with reference to the Kyoto Encyclopedia of Genes and Genomes (KEGG) database.

### 2.9. Detection of Key Metabolites in Serum and Intestine

The content of ethanol (ADS-W-FM030) and acetaldehyde (ADS-W-FM014-100) in the serum of rats was detected by a commercial kit. The contents of acetic acid (MM-926697O2) and acetyl-CoA (MM-71224R2) in serum were detected by ELISA. The contents of AA (MM-926637O2), LA (MM-928294O2), 13-HPODE (MM-928365O2), PGE2 (MM-0068R2), and LTB4 (MM-0048R2) in the small intestine of rats were detected by an ELISA kit. The kits were purchased from the Shanghai Majer BioEngineering Research Institute Co., Ltd. (Shanghai, China).

### 2.10. Quantitative Real-Time PCR

The detection and analysis methods followed established protocols [16]. After completion of the RT-qPCR reaction, the relative expression of target gene mRNA was calculated from the corresponding CT value, using β-actin as an internal reference. Primers were designed and synthesized by Qingdao Biotechnology Co., Ltd. (Beijing, China), and the primer sequences are shown in Table S3.

### 2.11. Western Blotting

Small intestinal tissue protein extracts were prepared and assessed using standard procedures. The primary antibody information is shown in Table S4. The gray value was analyzed using ImageJ v1.8.0 software.

### 2.12. Statistical Analysis

To eliminate potential bias, all researchers conducting histopathological assessments, biochemical tests, sequencing, and data interpretation remained unaware of group assignments during sample processing and initial data collection. A randomized coding system was implemented to conceal group identities, which were only disclosed after preliminary analyses were finalized. Statistical evaluations were carried out using IBM SPSS 19.0. For datasets with normal distribution and equal variance (confirmed by Levene’s test), one-way ANOVA, followed by Duncan’s post hoc analysis, was conducted. When normality was present but variance assumptions were violated, the Tamhane T2 post hoc test was used instead. GraphPad Prism 8.0.2 was utilized for data visualization. The results are presented as the mean ± standard deviation (SD), with each dataset replicated at least three times. *p* < 0.05 was considered statistically significant.

## 3. Results

### 3.1. Effect of LTA on Duodenal Pathological Damage and Permeability in Chronic Alcoholic Intestinal Injury Rats

The animal treatment and grouping in this study are shown in Figure 1A. The duodenum histological slices indicated that the MOD group (Figure 1B,C) exhibited intestinal villi with apical autolysis (blue arrow), inflammatory infiltration (red arrow), and mild lymphoid tissue hyperplasia of the submucosa (yellow arrow). Moreover, the histological scores of the MOD group were significantly increased compared to those in the CK group (Figure 1G, *p* < 0.01; the histological scoring criteria can be found in Supplementary Table S1). Administration of different LTA doses alleviated alcohol-induced pathological injury to the duodenum (Figure 1C–F). Compared with that of the MOD group, the histological score of the LTA200 group was significantly decreased (Figure 1G, *p* < 0.01). Compared with the LTA100 group, inflammatory infiltration was improved in the LTA200 and LTA400 groups (Figure 1D–F). The MOD group showed a significantly increase in serum DAO, D-LA, and LPS levels compared to the CK group (Figure 1H–J, *p* < 0.01). Compared to the MOD group, DAO, D-LA, and LPS levels were decreased in LTA groups.

### 3.2. LTA Ameliorates Inflammatory Infiltration and Oxidative Stress in the Intestine of Chronic Alcoholic Intestinal Injury Rats

**Figure 2:**
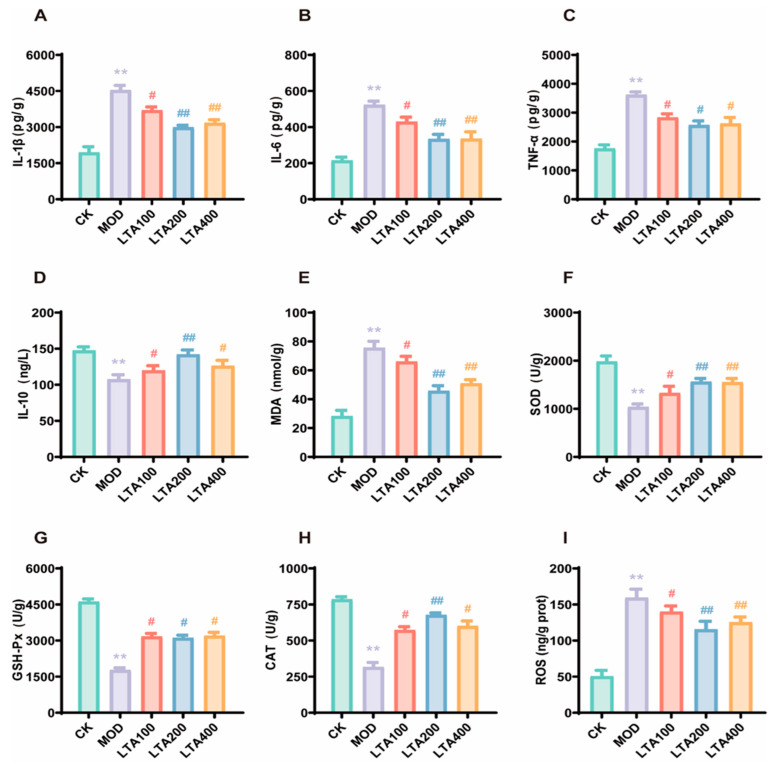
Effects of LTA on intestinal inflammatory factors and antioxidant capacity level in rats (n = 3). (**A**) interleukin-1β (IL-1β), (**B**) interleukin-6 (IL-6), (**C**) tumor necrosis factor-α (TNF-α), (**D**) interleukin-10 (IL-10), (**E**) malondialdehyde (MDA), (**F**) superoxide dismutase (SOD), (**G**) glutathione peroxidase-px (GSH-Px), (**H**) catalase (CAT), and (**I**) reactive oxygen species (ROS) levels. Compared with CK, **: *p* < 0.01; compared with MOD, #: *p* < 0.05, ##: *p* < 0.01. Data are presented as the mean ± SD.

To evaluate the anti-inflammatory properties of LTA, we measured concentrations of key cytokines—TNF-α, IL-1β, IL-6, and IL-10—in small intestinal tissue. The MOD group displayed substantially elevated IL-1β (Figure 2A), IL-6 (Figure 2B), and TNF-α (Figure 2C) levels (*p* < 0.01), alongside reduced IL-10 (Figure 2D) levels (*p* < 0.01), when compared to the CK group. However, LTA administration reversed these trends, lowering pro-inflammatory markers (IL-1β, IL-6, TNF-α) while boosting anti-inflammatory IL-10. The LTA200 group stood out, showing the most pronounced effects: significant decreases in IL-1β, IL-6, and TNF-α coupled with a sharp rise in IL-10. We also investigated LTA’s antioxidant capacity by analyzing intestinal tissue levels of GSH-Px, CAT, SOD, MDA, and ROS. The MOD group had significantly higher oxidative stress markers (MDA, Figure 2E; ROS, Figure 2I, *p* < 0.05) and diminished antioxidant enzyme activity (SOD, CAT, GSH-Px, Figure 2F–H, *p* < 0.05) relative to the CK group. LTA treatment countered these effects, reducing MDA and ROS while enhancing GSH-Px, CAT, and SOD activity. Once again, the LTA200 group outperformed the rest, with striking reductions in MDA (*p* < 0.01) and ROS (*p* < 0.01), alongside notable increases in CAT (*p* < 0.05), SOD (*p* < 0.01), and GSH-Px (*p* < 0.01).

These findings indicate that LTA effectively mitigates both inflammation and oxidative stress in chronic alcohol-induced intestinal damage, with the LTA200 dosage demonstrating the best effect.

### 3.3. Effects of LTA on Intestinal Microbiota in Rats with Chronic Alcoholic Intestinal Injury

**Figure 3:**
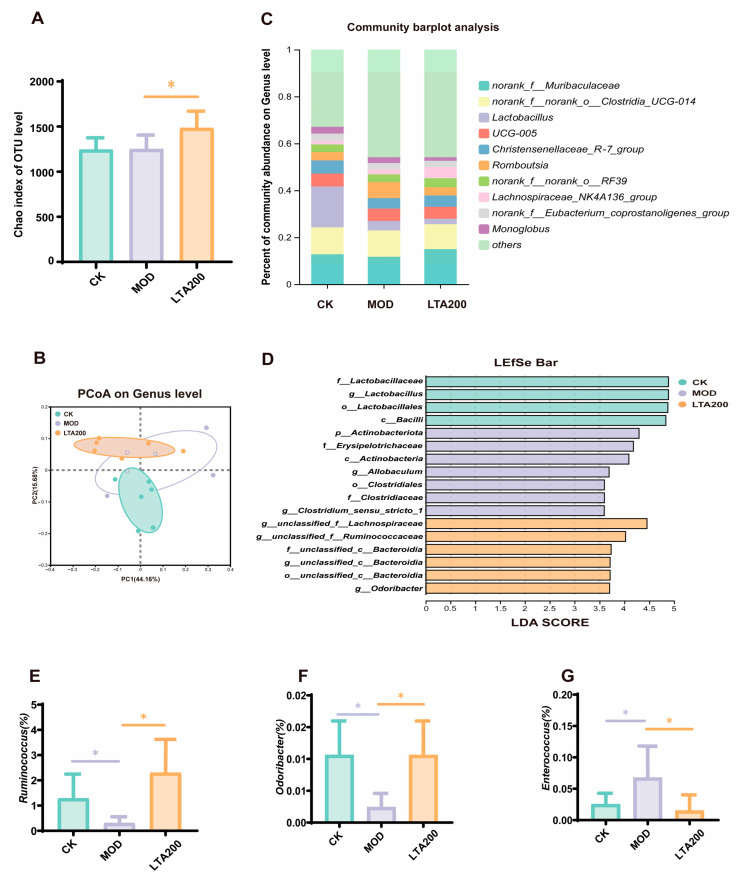
Effects of LTA on intestinal microbiota in rats (n = 6). (**A**) Chao index. (**B**) Principal component analysis (PCoA). (**C**) Genus-level community composition distribution. (**D**) LDA linear discriminant analysis. (**E**–**G**) The differences in microorganisms, including *Ruminococcus*, *Odoribacter*, and *Enterococcus*, between the CK, MOD, and LTA groups. Comparison between different groups, *: *p* < 0.05.

The Chao index was employed to evaluate the α diversity, revealing no significant difference between the CK and MOD groups. However, the Chao index for the LTA200 group was significantly elevated compared to the MOD group (Figure 3A, *p* < 0.05). These findings suggest that LTA intervention enhanced both the diversity and abundance of the overall microbiome. To examine structural changes in intestinal microbiota post-LTA intervention, principal coordinate analysis was conducted for β-diversity analysis (Figure 3B). Notably, the LTA200 and CK groups exhibited closer clustering compared to the MOD group, indicating that medium-dose theanine induced intestinal flora alterations akin to those seen in healthy rats. Community composition analysis at the genus level (Figure 3C) revealed that the intestinal flora was primarily composed of norank_f_Muribaculaceae (12.80%), norank_f__norank_o_Clostridia_ UCG-014 (11.47%), and Lactobacillus (17.40%). Additionally, Figure 3D shows that in group CK, Lactobacillus and Bacilli dominated. In contrast, the MOD group exhibited a predominance of Actinobacteriota and Clostridium_sensu_stricto_1. The LTA200 group was marked by significant levels of *Ruminococcus*, *Bacteroidia*, and *Odoribacter*. Significance testing revealed that the MOD group had lower abundances of *Ruminococcus* (Figure 3E, *p* < 0.05) and *Odoribacter* (Figure 3F, *p* < 0.05), alongside a higher abundance of *Enterococcus* compared to the CK group (Figure 3G, *p* < 0.05). Compared to the MOD group, LTA intervention significantly increased the abundance of *Ruminococcus* and *Odoribacter* while decreasing *Enterococcus* levels (*p* < 0.05), thereby enhancing intestinal environment stability.

### 3.4. Effects of LTA on Intestinal Metabolites in Rats with Chronic Alcoholic Intestinal Injury

**Figure 4:**
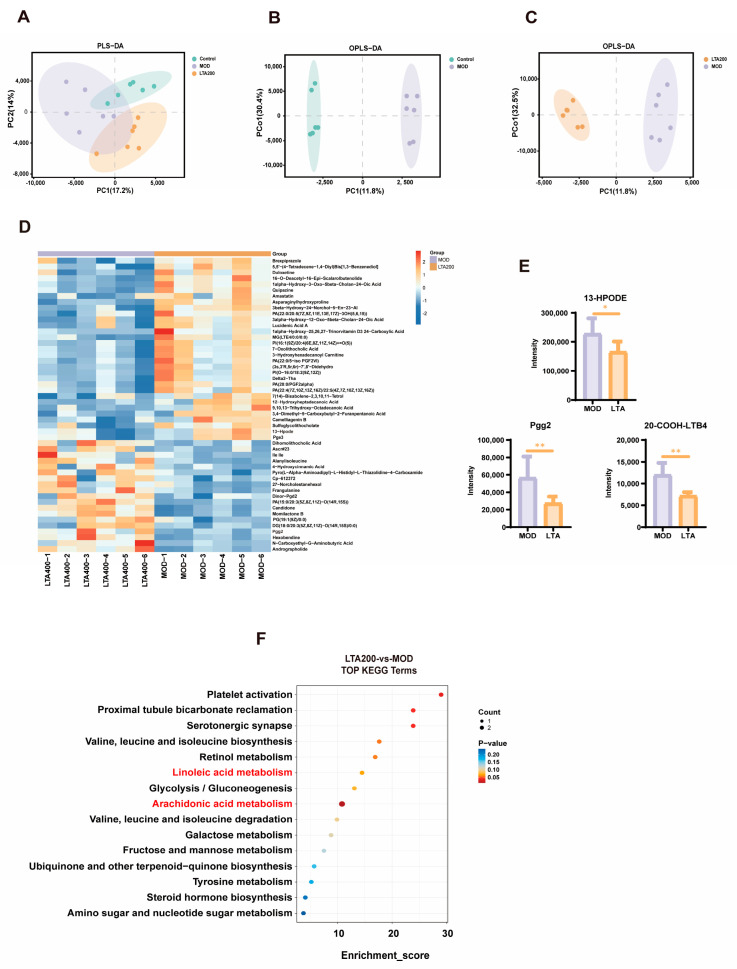
Effects of LTA on intestinal contents of metabolites in rats (n = 6). (**A**) Partial least squares discrimination analysis (PLS-DA) score plot. (**B**,**C**) Orthogonal partial least squares discrimination analysis (OPLS-DA) score plot. (**D**) Heat map of differential metabolite expression (LTA200 vs. MOD, TOP50). (**E**) Levels of three differential metabolites (PGG2, 13-HPODE, and 20-COOH-LTB4). (**F**) KEGG pathway classification (LTA200 vs. MOD). Comparison between different groups, *: *p* < 0.05, **: *p* < 0.01.

Untargeted metabolomics was performed on rat intestinal contents. The partial least squares discrimination analysis (PLS-DA, Figure 4A) and orthogonal partial least squares discrimination analysis (OPLS-DA) plots (Figure 4B,C) revealed that the CK, MOD, and LTA200 groups were clearly separated. The heat map demonstrates the differential metabolites of Top50 between the MOD and LTA200 groups (Figure 4D). We found a notable shift in metabolite levels, particularly involving amino acids, peptides, benzoic acids, bile acids, alcohols, carbohydrates, eicosanoids, and fatty acids. When comparing the LTA group to the MOD group, there was a marked decrease in the concentrations of 13-hydroperoxylinoleic acid (13-HPODE) (*p* < 0.05), prostaglandin G2 (PGG2), and 20-carboxy-leukotriene B4 (20-COOH-LTB4) (Figure 4E, *p* < 0.01). To pinpoint the key metabolic pathways influenced by these changes, we enriched intestinal metabolites in KEGG and found that the results focused on several pathways (LTA200 vs. MOD), including arachidonic acid (AA) and linoleic acid (LA) metabolism, glycolysis/gluconeogenesis, degradation of branched-chain amino acids (valine, leucine, and isoleucine), tyrosine metabolism, amino sugar and nucleotide sugar metabolism, among other relevant signaling pathways (Figure 4F). Notably, the LTA200 group exhibited significantly elevated levels of 13-HPODE, PGG2, and 20-COOH-LTB4 within AA and LA metabolic pathways compared to the MOD group. These findings highlight the substantial impact of LTA on these specific biochemical processes.

### 3.5. LTA Regulates mRNA Expression of Intestinal Alcohol Metabolism and the LA-AA Metabolic Pathway

**Figure 5:**
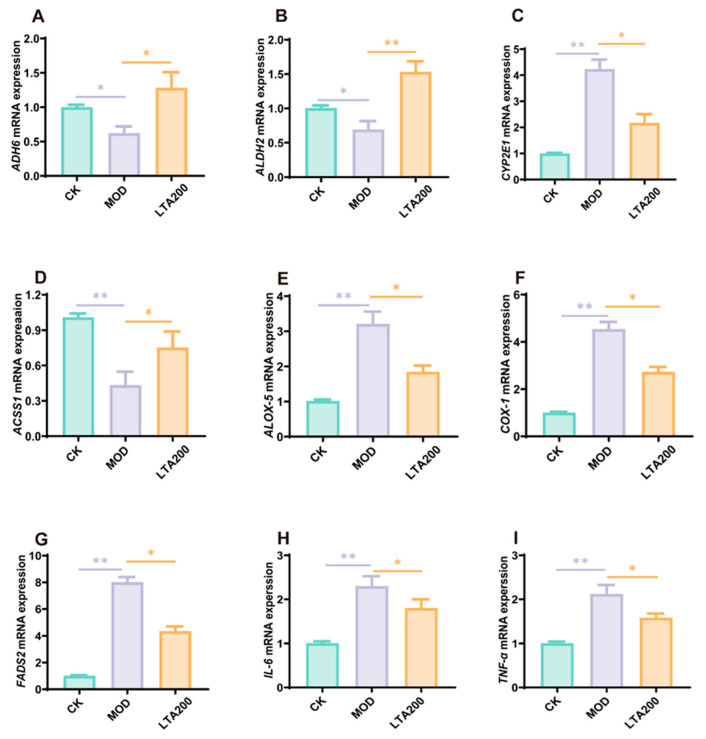
Effects of LTA on the expression of key mRNA in intestinal alcohol metabolism and LA-AA metabolism (n = 3). (**A**) *ADH6*. (**B**) *ALDH2*. (**C**) *CYP2E1*. (**D**) *ACSS1*. (**E**) *ALOX-5*. (**F**) *COX-1*. (**G**) *FADS2*. (**H**) *IL-6*. (**I**) *TNF-α*. Comparison between different groups, *: *p* < 0.05, **: *p* < 0.01.

We quantified the mRNA expression of key alcohol-metabolizing enzymes—ADH6, ALDH2, CYP2E1, and ACSS1—in small intestinal tissue. Compared to the CK group, the MOD group exhibited significantly reduced expression of ADH6 (*p* < 0.05), ALDH2 (*p* < 0.05), and ACSS1 (*p* < 0.01), while CYP2E1 showed increased expression (Figure 5A–C, *p* < 0.01). Treatment with LTA reversed these trends, elevating ADH6 (*p* < 0.05), ALDH2 (*p* < 0.01), and CYP2E1 (*p* < 0.05) while suppressing ACSS1 (*p* < 0.05) relative to the MOD group. Further analysis confirmed altered mRNA expression in the LA-AA metabolic pathway. The MOD group displayed elevated levels of ALOX-5, COX-1, and FADS2 compared to controls (Figure 5E–G, *p* < 0.01). However, LTA treatment counteracted this effect, significantly lowering the expression of all three enzymes (*p* < 0.05). Additionally, we assessed pro-inflammatory cytokine expression in the small intestine. The MOD group showed marked upregulation of IL-6 and TNF-α mRNA levels versus the CK group (Figure 5H,I, *p* < 0.01). LTA intervention mitigated this inflammatory response, reducing both IL-6 (*p* < 0.05) and TNF-α (*p* < 0.05) expression compared to the MOD group.

### 3.6. LTA Regulates the Expression of Key Proteins and Metabolites in the Alcohol and LA-AA Metabolic Pathways in the Intestines

**Figure 6:**
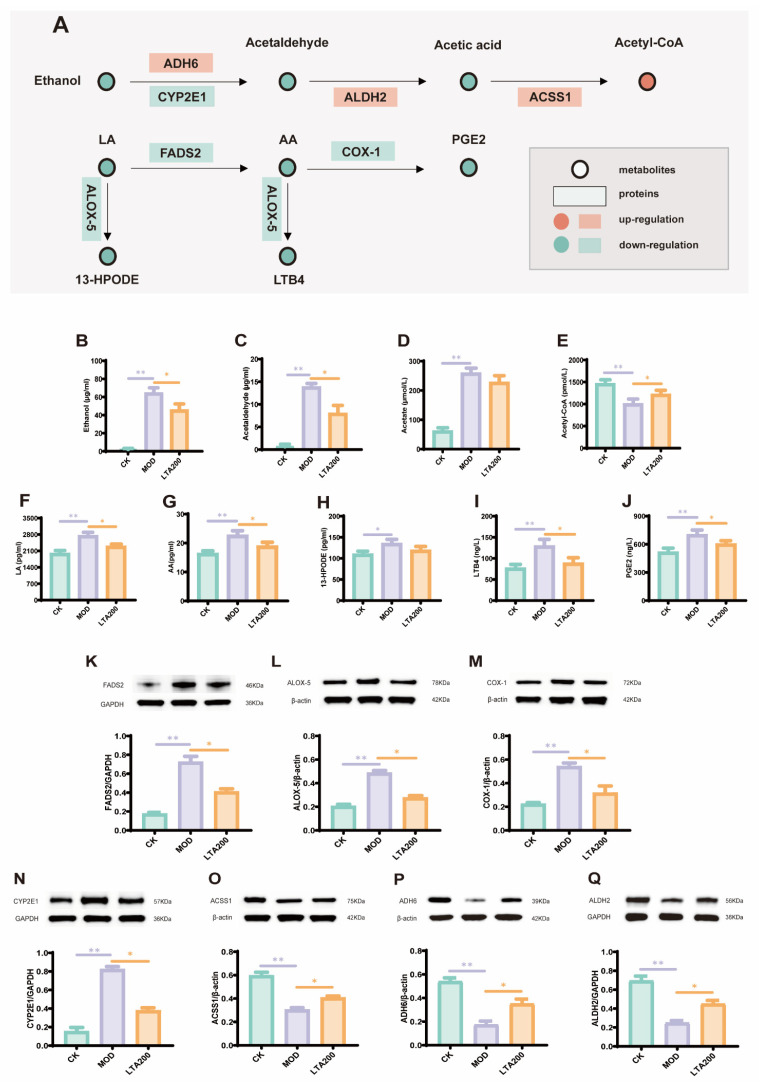
Effects of LTA on the expression of key proteins (n = 3) and metabolites (n = 6) in intestinal alcohol metabolism and LA-AA metabolism. (**A**) Schematic diagram of key proteins and metabolites in alcohol metabolism and arachidonic acid–linoleic acid metabolism regulated by LTA. (**B**–**E**) Serum levels of ethanol, acetaldehyde, acetic acid, and acetyl-CoA. (**F**–**J**) Metabolite levels of LA, AA, 13-HPODE, LTB4, and PGE2 in intestinal tissue. (**K**–**Q**) Protein levels of FADS2, ALOX-5, COX-1, CYP2E1, ACSS1, ADH6, and ALDH2 in intestinal tissue. Comparison between different groups, *: *p* < 0.05, **: *p* < 0.01.

The levels of metabolites and proteins associated with alcohol metabolism as well as those related to the LA-AA metabolic pathway were further analyzed. The results of metabolite detection indicated that, compared with those in the CK group, the MOD group exhibited significantly elevated levels of ethanol, acetaldehyde, acetic acid, LA, AA, PGE2, 13-HPODE, and LTB4 (Figure 6B–D,F–J, *p* < 0.01), whereas acetyl-CoA levels were significantly reduced (Figure 6E, *p* < 0.01). Unlike the MOD group, the LTA200 group demonstrated a significant decrease in the levels of ethanol, acetaldehyde, LA, AA, PGE2, 13-HPODE, and LTB4 (*p* < 0.01), along with a significant increase in acetyl-CoA levels (*p* < 0.01). Notably, acetic acid content did not change significantly in the LTA200 and MOD groups.

The protein detection results indicated that the levels of ADH6, ALDH2, and ACSS1 proteins were significantly downregulated (Figure 6O–Q, *p* < 0.01), and those of CYP2E1, COX-1, ALOX-5, and FADS2 proteins were significantly upregulated in the MOD group, compared with the CK group (Figure 6K–N, *p* < 0.01). Relative to those in the MOD group, the levels of ADH6, ALDH2, and ACSS1 were upregulated, and those of CYP2E1, COX-1, ALOX-5, and FADS2 were downregulated in the LTA200 group.

## 4. Discussion

In this study, we investigated the protective effects and underlying mechanisms of LTA in the intestines of SD rats subjected to chronic alcohol consumption. Previous studies have shown that intestinal injury mainly involves pathological injury to the intestinal tissue, permeability changes, inflammatory response, oxidative stress, flora, and metabolic level disturbances [28,29]. In the present study, we successfully established a model of chronic alcoholic intestinal injury by evaluating intestinal pathological injury, permeability, inflammatory factors, and oxidative stress levels [30,31]. The results indicated that long-term alcohol intake would lead to pathological damage of the duodenum in rats, increase intestinal permeability, and trigger intestinal inflammation and oxidative imbalance, suggesting that chronic alcohol intake could cause intestinal damage and the successful establishment of a chronic alcohol-induced intestinal injury model [29,32].

To investigate whether LTA can protect against chronic alcohol-induced intestinal injury, rats were fed with LTA. The results indicated that LTA reduced the pathological injury of duodenal and intestinal permeability in chronic alcoholic rats. Similar effects have been observed in other studies using different models [16,33]. The accumulation of ROS often leads to oxidative stress in the intestine [34]. Previous studies have reported that alcohol consumption increases MDA and ROS levels in the intestine and decreases intestinal GSH-Px, SOD, and CAT activities [35,36], leading to chronic intestinal injury, which is consistent with our findings. It is worth noting that LTA intervention significantly attenuated oxidative stress in intestinal tissues, as evidenced by reduced ROS and MDA levels alongside elevated SOD, GSH-Px, and CAT activities. We assessed levels of gut inflammatory factors, which are a proxy for gut health [37,38]. Specifically, chronic alcohol intake increased the levels of pro-inflammatory cytokines (IL-1β, IL-6, TNF-α) and decreased the level of anti-inflammatory cytokine IL-10 in the intestine of rats. LTA intervention restored these indicators. Notably, a similar efficacy of LTA was also shown in mice with ulcerative colitis [11,39]. Therefore, LTA protects against chronic alcoholic intestinal injury, and the best effect was demonstrated in the LTA200 group. Normally, higher doses of biologically active compounds are associated with enhanced activity, but nonlinear dose-response relationships are not uncommon in nutritional and pharmacologic interventions. It is hypothesized that this phenomenon arises because the strong upregulation of IL-10 by LTA200 may reflect an optimal immunomodulatory balance. However, LTA400 may over-suppress inflammatory signals, inadvertently impair host defense mechanisms or tissue remodeling processes critical for recovery, as similarly described in an overcompensation study of anti-inflammatory effects [40].

We used 16S high-throughput sequencing to explore the effects of LTA on intestinal microbiota. α and β diversities reflect the abundance and composition of the intestinal flora [41]. Our results showed that although the Chao index was similar between the MOD and CK groups, PCoA showed a significant separation of the colony structure between the two, suggesting an overall shift in colony composition due to alcohol. Notably, alcohol consumption did not induce significant alterations in α diversity. The Chao index mainly reflects species richness but is not sensitive to evenness. Chronic alcohol may preferentially lead to a “trade-off” between specific genera rather than a significant change in the overall number of species. Similarly, in a study examining the effects of long-term alcohol consumption on gut microbiota, no significant changes in α diversity were observed [11]. Thus, we analyzed the changes in specific flora between groups; specific groups of microbes in the intestine have potential impacts on intestinal health. As beneficial bacteria in the intestinal tract, *Ruminococcus* and *Odoribacter* play significant roles in ameliorating intestinal inflammation [42]. In contrast, *Enterococcus* is considered a harmful bacterium, and its increased abundance is often linked to gut inflammation [43]. In our study, we also observed that following LTA intervention, the abundance of *Ruminococcus* and *Odoribacter* increased, whereas that of *Enterococcus* decreased.

We analyzed the changes in metabolites of intestinal content and discovered that LTA significantly inhibited the production of PGG2, 20-COOH-LTB4, and 13-HOPDE within the LA and AA metabolic pathways. Notably, all of these have potential pro-inflammatory effects on intestinal inflammation [44,45,46]. Specifically, 13-HPODE is a reactive oxygen species-derived metabolite generated by oxidation of LA by LOX, which can directly disrupt the membrane integrity of intestinal epithelial cells, activate the NF-κB pathway, and induce the release of pro-inflammatory factors, such as IL-6 and TNF-α [45]. PGE2, which is generated by COX-1-catalyzed AA, can promote the release of IL-6 and TNF-α and exacerbate inflammation [15]. LTB4, catalyzed by ALOX-5 for AA production, is a potent chemokine that promotes neutrophil infiltration and releases ROS and proteases, which directly lead to apoptosis of intestinal epithelial cells [47]. KEGG pathway enrichment analysis revealed that LTA intervention affected LA and AA metabolism. Thus, it is reasonable to speculate that LTA protects against intestinal inflammatory injury by regulating LA-AA metabolism. Subsequently, we analyzed the key mRNAs, proteins, and metabolites in the LA-AA metabolic pathway. As expected, LTA decreased the mRNA and protein expression levels of FADS2, COX-1, and ALOX-5 while decreasing those of LA, AA, PGE2, 13-HPODE, and LTB4 relative to those in the MOD group. In addition, LTA reduced the IL-6 and TNF-α mRNA levels.

Unlike in ulcerative colitis [11] or heat stress models [23], in this study, the function of LTA in maintaining intestinal health also includes its impact on alcohol metabolism, and understanding the role of acetaldehyde in intestinal alcohol metabolism is crucial for the development of strategies to mitigate alcohol-induced intestinal injury. Previous studies have demonstrated elevated levels of acetaldehyde in the intestinal lumen after alcohol administration [48]. Furthermore, intestinal ALDH2 has been identified as a key enzyme facilitating the clearance of acetaldehyde both within the intestine and throughout the body [3]. Notably, our previous research revealed that LTA can enhance hepatic alcohol metabolism, thereby accelerating acetaldehyde clearance. Therefore, we speculated that LTA may boost intestinal alcohol metabolism, thereby preventing chronic alcohol-induced intestinal injury. We further analyzed key mRNAs, proteins, and metabolites involved in alcohol metabolism. As expected, LTA reduced the levels of acetaldehyde and acetic acid caused by chronic alcohol consumption while increasing acetyl-CoA levels. In addition, the mRNA and protein expression levels of ADH6, ALDH2, and ACSS1 were upregulated by LTA intervention, whereas CYP2E1 expression was downregulated.

In summary, the underlying protective mechanism of LTA against chronic alcoholic intestinal injury may involve the acceleration of intestinal alcohol metabolism, which helps reduce oxidative stress in the intestines. It may also involve regulating LA-AA metabolism to mitigate inflammatory responses in the intestinal environment. However, this study mainly emphasizes the overall role of microorganisms, and the contribution of individual microbial species in mitigating intestinal injury is not clear. Therefore, germ-free or gnotobiotic rats can be used in subsequent studies to analyze the involvement of the microbiota and to validate the main results of this study. Moreover, in vitro validation or FMT experiments can further verify the contribution of microbiota in the mechanism of LTA, which will be our next research direction. Because of the co-metabolism between the host and intestinal microbiota, this study did not clarify the sources of the differential metabolites. Due to the interconnectedness of the mechanisms, whether the modulation of LA-AA metabolism is a result of reduced oxidative stress or a direct effect of LTA is unproven; future studies could validate this through in vitro cell modeling experiments or knockout experiments.

## 5. Conclusions

In this study, LTA can reduce intestinal pathological damage, intestinal oxidative stress, and inflammatory damage. At the same time, LTA can regulate the structure of intestinal flora and maintain the homeostasis of intestinal flora, and the optimal dose is 200 mg·kg^−1^·d^−1^. In addition, LTA regulates intestinal alcohol and LA-AA metabolism and upregulates the gene and protein expression levels of ADH6, ALDH2, and ACSS1 while downregulating those of CYP2E1, FADS2, ALOX-5, and COX-1. Concurrently, LTA increases the levels of metabolites, such as acetyl-CoA, while decreasing those of ethanol, acetaldehyde, acetic acid, LA, AA, PGE2, 13-HPODE, and LTB4. In conclusion, our findings demonstrated that LTA can mitigate chronic alcoholic intestinal injury by modulating intestinal alcohol and LA-AA metabolism. This study provides a new strategy for the prevention and treatment of alcoholic intestinal injury and provides a scientific basis for LTA as a dietary supplement.

## Data Availability

The data are not publicly available due to privacy, and data supporting the results are available from the corresponding author upon reasonable request.

## Supplementary Materials

The following supporting information can be downloaded at: https://www.mdpi.com/article/10.3390/nu17111943/s1, Figure S1. Evolution of body weight and food intake of SD rats during the course of the feeding. Table S1. Main Reagents. Table S2. Histological scoring criteria. Table S3. Related gene primer sequences. Table S4. Specific information on primary and secondary antibodies of Western blot.
