# Supplementary material for: L-Theanine Mitigates Chronic Alcoholic Intestinal Injury by Regulating Intestinal Alcohol and Linoleic-Arachidonic Acid Metabolism in Rats
Source: Nutrients. 2025 Jun 5;17(11):1943. doi: 10.3390/nu17111943 (PMC12157802; doi:10.3390/nu17111943)
Supplement: Supplementary file 1 [file nutrients-17-01943-s001.zip › Supplementary material1.pdf]

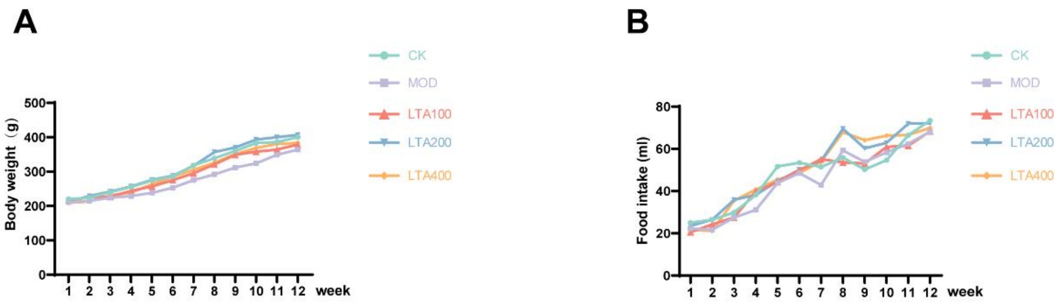

Figure S1. Evolution of body weight and food intake of SD rats during the course of the feeding.

Table S1 Main Reagents

Name	Manufacturer
LTA (>98% purity)	Hunan Sanfu Biotechnology Co., Ltd. (Changsha, China).
Lieber-DeCarli diet	Hunan Slack Jingda Laboratory Animal Co., Ltd. (Changsha, China)

Table S2 Histological scoring criteria

scroe	inflammation	lymphoid tissue	intestinal villus	Intestinal gland
0	none	none	none	none
1	occasional	occasionally seen lymphoid follicles	distorted and deformed intestinal villi	irregular morphology of intestinal glands
2	inflammation of submucosa cells	mild hyperplasia of lymphatic tissue	shortening of small intestinal villi	reduced number of intestinal glands
3	inflammation of the submucosa and lamina propria	severe hyperplasia of lymphatic tissue	large amount of intestinal villi becoming shorter	
4	extensive inflammation of the submucosa and lamina propria		intestinal villus autolysis	

Table S3 Related gene primer sequences

Gene Name	Forward nucleotide sequence primers (5'-3')	Reverse nucleotide sequence primers (5 '-3')
ADH6	CGGCTTGTCTGTTGTCATGG	CTCTCATTGCAGGAGGCGAG
ALDH2	CTATACCCGCCATGAGCCTG	GAACGCCACTTTGTCCACAC
CYP2E1	TGGTCCTGCATGGCTACAAG	CGGGCCTCATTACCCTGTTT
ACSS1	ACCCTGATGCTGGTCGTTAC	GGCTTCGTGGTTGATAGGCT
ALOX-5	AGCTGTGCATGGCCTCTTGT	CCAATGTCAATGGCAACACC
FADS2	TGGCAAAGTGAATGGCAAGC	TCATAGTGGTCAGGGTCCGT
COX-1	GAGCCCCCTAGTGATGTGTG	AGACCAACCGTCAGGAGTCA
IL-6	CCTGGAGTTTGTGAAGAACA	GGAAGTTGGGGTAGGAAGGA
TNF-α	CCCTCACACTCACAAACCAC	ACAAGGTACAACCCATCGGC
β-actin	GGCTGTATTCCCTCCATCG	CCAGTTGGTAACAATGCCATG

Table S4 Specific information on primary and secondary antibodies of Western blot

Name	Source of antibody	Dilution ratio	molecular weight
ADH6	affinity	1: 1000	39 KDa
ALDH2	affinity	1: 1000	56 KDa
COX-1	proteintech	1: 5000	72 KDa
CYP2E1	affinity	1: 1000	57 KDa
ALOX-5	affinity	1: 1000	78 KDa
ACSS1	affinity	1: 1000	75 KDa
FADS2	affinity	1: 5000	46 KDa
β-actin	proteintech	1: 10000	42 KDa
GAPDH	proteintech	1: 10000	36 KDa
HRP-Goat anti-Rabbit	Abiowell	1: 5000	——
